# 3-{5-Bromo-2-[(tri­phenyl­phosphanyl­idene)amino]­phen­yl}-4,5-di­hydro-1,2,3-oxa­diazol-3-ylium-5-olate

**DOI:** 10.1107/S1600536813017765

**Published:** 2013-07-03

**Authors:** David Grossie, Leanna Harrison, Kenneth Turnbull

**Affiliations:** a Department of Chemistry, Wright State University, Dayton, OH 45435, USA

## Abstract

In general, sydnone compounds are synthesized with an aromatic substituent at the N-3 position and this feature adds to the stability of the mesoionic five-membered heterocyclic ring. In the title compound, C_26_H_19_BrN_3_O_2_P, the aromatic substitutent is tri­phenyl­phosphine 4-bromo­phenyl­imide. The dihedral angle between the planes of the sydnone and the attached phenyl ring is 45.98 (7)°. In the crystal, the mol­ecules packed as pairs in which the sydnone rings lie in parallel planes separated by 0.849 Å and sandwiched between two parallel phenyl rings. The mol­ecules inter­act through cyclic C—H⋯O=C hydrogen bonds.

## Related literature

For more information on the sydnone family of compounds, see: Ohta & Kato (1969 ▶). For their synthesis and structures, see: Grossie & Turnbull (1992 ▶); Grossie *et al.* (2001 ▶, 2007 ▶); Hope & Thiessen (1969 ▶); Hodson & Turnbull (1985 ▶); Ollis & Ramsden (1976 ▶); Riddle *et al.* (2004*a* ▶,*b* ▶,*c* ▶).
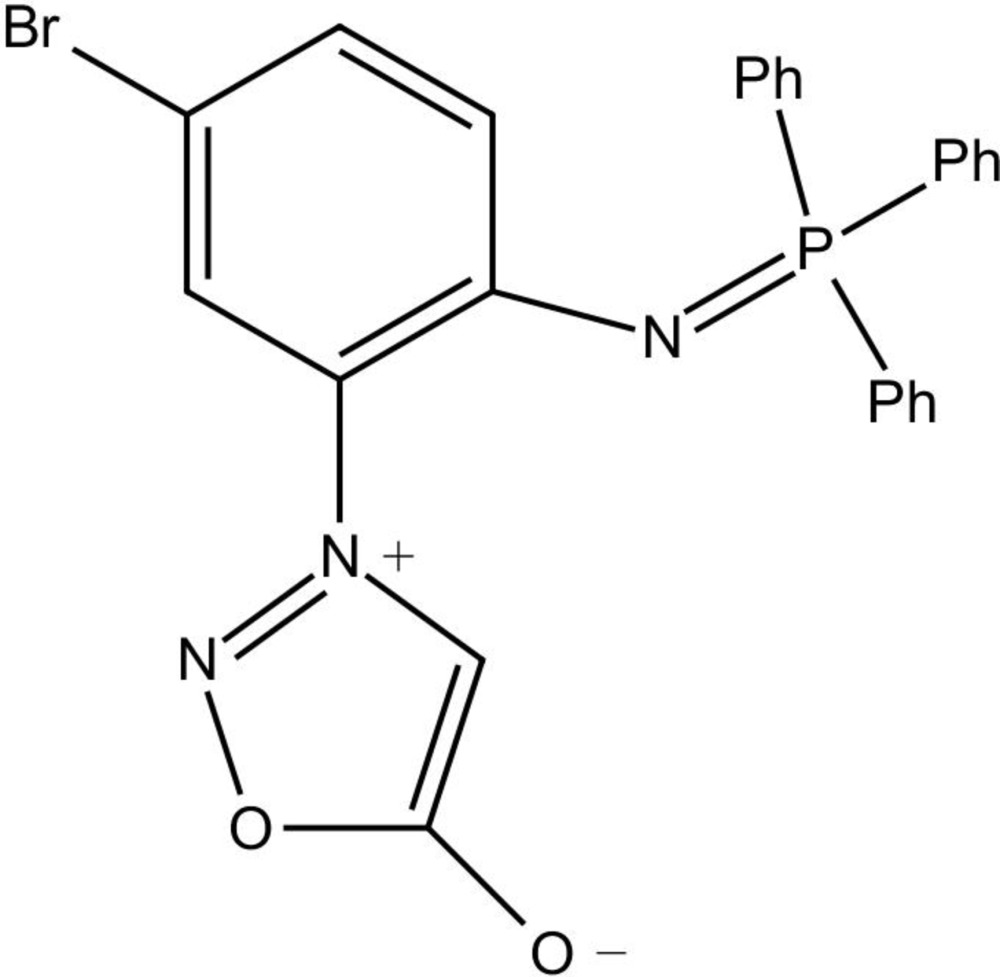


## Experimental

### 

#### Crystal data

C_26_H_19_BrN_3_O_2_P
*M* *_r_* = 516.33
Monoclinic, 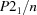

*a* = 7.5207 (8) Å
*b* = 13.8672 (15) Å
*c* = 21.816 (2) Å
β = 95.449 (2)°
*V* = 2264.9 (4) Å^3^
*Z* = 4
Mo *K*α radiation
μ = 1.92 mm^−1^
*T* = 173 K
0.43 × 0.30 × 0.28 mm

#### Data collection

Bruker Kappa APEXII diffractometer
Absorption correction: multi-scan (*SADABS*; Siemens, 1996 ▶) *T*_min_ = 0.50, *T*_max_ = 0.58
46513 measured reflections
6964 independent reflections
5914 reflections with *I* > 2.0σ(*I*)
*R*_int_ = 0.029

#### Refinement

*R*[*F* ^2^ > 2σ(*F* ^2^)] = 0.029
*wR*(*F* ^2^) = 0.074
*S* = 0.92
6964 reflections
298 parameters
H-atom parameters constrained
Δρ_max_ = 0.86 e Å^−3^
Δρ_min_ = −0.48 e Å^−3^

### 

Data collection: *APEX2* (Bruker, 2006 ▶); cell refinement: *SAINT* (Bruker, 2006 ▶); data reduction: *SAINT*; program(s) used to solve structure: *SIR92* (Altomare *et al.*, 1994 ▶); program(s) used to refine structure: *CRYSTALS* (Betteridge *et al.*, 2003 ▶); molecular graphics: *CAMERON* (Watkin *et al.*, 1996 ▶); software used to prepare material for publication: *CRYSTALS*.

## Supplementary Material

Crystal structure: contains datablock(s) global, I. DOI: 10.1107/S1600536813017765/gg2120sup1.cif

Structure factors: contains datablock(s) I. DOI: 10.1107/S1600536813017765/gg2120Isup2.hkl

Supplementary material file. DOI: 10.1107/S1600536813017765/gg2120Isup3.cml

Additional supplementary materials:  crystallographic information; 3D view; checkCIF report

## Figures and Tables

**Table 1:** Hydrogen-bond geometry (Å, °)

*D*—H⋯*A*	*D*—H	H⋯*A*	*D*⋯*A*	*D*—H⋯*A*
C4—H41⋯O5^i^	0.91	2.48	3.344 (2)	159
C72—H721⋯O5^i^	0.94	2.37	3.297 (2)	173

Symmetry code: (i) 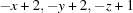.

**Fig. 1.**
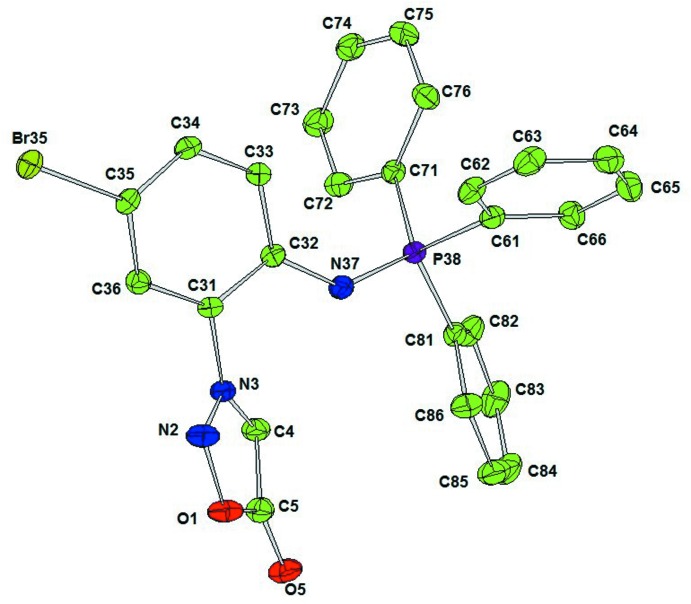
The title compound with displacement ellipsoids drawn at the 50% probability level. H atoms are shown as spheres of arbitary radius.

## supplementary crystallographic information

### Comment

In the title compound the bond distances and angles are within the expected ranges. The sydnone ring (O1– C5) and the phenyl ring (C31 – C36) in the structure are planar, all deviations from the mean plane being less than 0.1 Å. The angle between the planes of the sydnone and the attached phenyl ring is 45.98°. The phenyl ring containing the bromine is stacked through the unit cell in a herringbone pattern in the b direction of the unit cell with slippage in the stack of 4.466 Å. The molecules are oriented in an alternating pattern in these herringbone stacks. The analysis of short ring interactions shows a distance between the sydnone and the phenyl ring (C31 – C36) in a symmetry related molecule as 5.8852 (1) Å. A H - π-ring interaction between C(33) and H(331) has a distance of 2.68 Å. The analysis of the *X*—Y *Cg*(Pi-Ring) interactions show that the interaction between the sydnone ring and Br(35) have a *X*···*Cg* of 3.9267 Å. The bromine atom shows flattening in the direction of the bond to C(35). The analysis of short intra and inter-molecular forces reveals multiple contacts within the structure, two of which have parameters suggestive of hydrogen bonding.

### Experimental

Triphenylphosphine-2-(4-bromo-3-sydnonyl)phenyl imide was prepared from 3-(2-amino-5-bromophenyl)sydnone with an 85% yield *via* a Mitsunobu process involving treatment with triphenylphosphine (1.1 eq) then diisopropyl azodicarboxylate (1.1 eq) in dry tetrahydrofuran at room temperature for 4 h.

### Refinement

The H atoms were all located in a difference map, but those attached to carbon atoms were repositioned geometrically. The H atoms were initially refined with soft restraints on the bond lengths and angles to regularize their geometry (C—H in the range 0.93–0.98, N—H in the range 0.86–0.89 N—H to 0.86 O—H = 0.82 Å) and *U*_iso_(H) (in the range 1.2–1.5 times *U*_eq_ of the parent atom), after which the positions were refined with riding constraints.

### Crystal data

**Table:**

C_26_H_19_BrN_3_O_2_P	*F*(000) = 1048
*M**_r_* = 516.33	*D*_x_ = 1.514 Mg m^−^^3^
Monoclinic, *P*2_1_/*n*	Mo *K*α radiation, λ = 0.71073 Å
Hall symbol: -P 2yn	Cell parameters from 8702 reflections
*a* = 7.5207 (8) Å	θ = 5–60°
*b* = 13.8672 (15) Å	µ = 1.92 mm^−^^1^
*c* = 21.816 (2) Å	*T* = 173 K
β = 95.449 (2)°	Block, yellow
*V* = 2264.9 (4) Å^3^	0.43 × 0.30 × 0.28 mm
*Z* = 4	

### Data collection

**Table:**

Bruker Kappa APEXII diffractometer	5914 reflections with *I* > 2.0σ(*I*)
Graphite monochromator	*R*_int_ = 0.029
ω scans	θ_max_ = 31.3°, θ_min_ = 1.7°
Absorption correction: multi-scan (*SADABS*; Siemens, 1996)	*h* = −10→10
*T*_min_ = 0.50, *T*_max_ = 0.58	*k* = −19→19
46513 measured reflections	*l* = −30→30
6964 independent reflections	

### Refinement

**Table:**

Refinement on *F*^2^	Primary atom site location: structure-invariant direct methods
Least-squares matrix: full	Hydrogen site location: inferred from neighbouring sites
*R*[*F*^2^ > 2σ(*F*^2^)] = 0.029	H-atom parameters constrained
*wR*(*F*^2^) = 0.074	Method = Modified Sheldrick *w* = 1/[σ^2^(*F*^2^) + ( 0.04*P*)^2^ + 1.42*P*] , where *P* = (max(*F*_o_^2^,0) + 2*F*_c_^2^)/3
*S* = 0.92	(Δ/σ)_max_ = 0.002
6964 reflections	Δρ_max_ = 0.86 e Å^−^^3^
298 parameters	Δρ_min_ = −0.48 e Å^−^^3^
0 restraints	

### Special details

**Table:**

Geometry. Least-squares planes (*x*,*y*,*z* in crystal coordinates) and deviations from them (* indicates atom used to define plane)Sydnone ring0.7299 (5) *x* - 0.6166 (6) *y* + 0.2950 (7) *z* = -0.190 (11)* -0.006 (1) O1 * 0.004 (1) N2 * 0.001 (1) N3 * -0.007 (1) C4 * 0.008 (1) C5Phenyl ring at N(3)-0.4830 (5) *x* + 0.7617 (4) *y* + 0.4318 (5) *z* = 10.247 (6)* -0.007 (1) C31 * 0.003 (1) C32 * 0.004 (1) C33 * -0.008 (1) C34 * 0.004 (1) C35 * 0.004 (1) C36 * 0.066 (1) Br35Angle to previous plane (with approximate e.s.d.) = 45.99 (7)

### Fractional atomic coordinates and isotropic or equivalent isotropic displacement parameters (Å^2^)

**Table:**

	*x*	*y*	*z*	*U*_iso_*/*U*_eq_	
Br35	0.226245 (18)	0.622503 (11)	0.439272 (7)	0.0272	
C35	0.45423 (17)	0.65812 (10)	0.47685 (6)	0.0192	
C36	0.55726 (17)	0.72268 (9)	0.44721 (6)	0.0179	
C31	0.72208 (16)	0.74960 (9)	0.47645 (6)	0.0159	
N3	0.83407 (14)	0.81101 (8)	0.44354 (5)	0.0172	
C4	0.93052 (19)	0.88673 (10)	0.46540 (7)	0.0209	
C5	1.02656 (19)	0.91890 (10)	0.41683 (7)	0.0233	
O5	1.13573 (15)	0.98178 (8)	0.41029 (6)	0.0315	
O1	0.97050 (15)	0.85637 (8)	0.36779 (5)	0.0261	
N2	0.85054 (17)	0.78868 (9)	0.38551 (6)	0.0231	
C32	0.78948 (17)	0.71586 (9)	0.53535 (6)	0.0165	
N37	0.95384 (15)	0.74604 (8)	0.55906 (5)	0.0194	
P38	1.05534 (4)	0.74521 (2)	0.625347 (15)	0.0158	
C81	1.21291 (17)	0.84316 (9)	0.62612 (6)	0.0177	
C82	1.22728 (19)	0.91448 (11)	0.67099 (7)	0.0248	
C83	1.3422 (2)	0.99241 (12)	0.66550 (8)	0.0301	
C84	1.44642 (19)	0.99724 (11)	0.61692 (8)	0.0290	
C85	1.4353 (2)	0.92545 (11)	0.57256 (8)	0.0280	
C86	1.3175 (2)	0.84922 (10)	0.57645 (7)	0.0238	
C61	1.18310 (17)	0.63657 (9)	0.64233 (6)	0.0180	
C62	1.12610 (18)	0.55137 (10)	0.61256 (7)	0.0223	
C63	1.2185 (2)	0.46591 (11)	0.62551 (8)	0.0264	
C64	1.3693 (2)	0.46523 (11)	0.66730 (7)	0.0265	
C65	1.4274 (2)	0.54983 (11)	0.69687 (7)	0.0263	
C66	1.33492 (19)	0.63547 (10)	0.68462 (7)	0.0227	
C71	0.91867 (17)	0.76109 (10)	0.68830 (6)	0.0179	
C72	0.81337 (19)	0.84392 (10)	0.68936 (7)	0.0225	
C73	0.6995 (2)	0.85532 (11)	0.73560 (7)	0.0260	
C74	0.68674 (19)	0.78413 (11)	0.77958 (6)	0.0247	
C75	0.7890 (2)	0.70123 (12)	0.77832 (7)	0.0272	
C76	0.90552 (19)	0.68980 (11)	0.73294 (7)	0.0244	
C33	0.67735 (17)	0.65022 (10)	0.56305 (6)	0.0190	
C34	0.51400 (18)	0.62114 (10)	0.53432 (6)	0.0196	
H361	0.5190	0.7485	0.4081	0.0233*	
H41	0.9287	0.9095	0.5044	0.0256*	
H821	1.1602	0.9097	0.7057	0.0301*	
H831	1.3474	1.0416	0.6953	0.0364*	
H841	1.5233	1.0489	0.6139	0.0343*	
H851	1.5054	0.9288	0.5384	0.0345*	
H861	1.3078	0.8016	0.5461	0.0291*	
H621	1.0273	0.5523	0.5829	0.0268*	
H631	1.1804	0.4086	0.6056	0.0317*	
H641	1.4308	0.4073	0.6754	0.0314*	
H651	1.5269	0.5494	0.7255	0.0320*	
H661	1.3731	0.6927	0.7041	0.0277*	
H721	0.8187	0.8918	0.6593	0.0263*	
H731	0.6310	0.9122	0.7360	0.0311*	
H741	0.6102	0.7900	0.8110	0.0298*	
H751	0.7780	0.6529	0.8082	0.0338*	
H761	0.9720	0.6334	0.7326	0.0299*	
H331	0.7152	0.6245	0.6017	0.0224*	
H341	0.4431	0.5785	0.5532	0.0248*	

### Atomic displacement parameters (Å^2^)

**Table:**

	*U*^11^	*U*^22^	*U*^33^	*U*^12^	*U*^13^	*U*^23^
Br35	0.01877 (7)	0.03313 (9)	0.02875 (8)	−0.00838 (5)	−0.00334 (5)	0.00226 (6)
C35	0.0152 (5)	0.0202 (6)	0.0220 (6)	−0.0033 (5)	0.0010 (4)	−0.0017 (5)
C36	0.0188 (6)	0.0182 (6)	0.0167 (5)	−0.0006 (5)	0.0014 (4)	−0.0003 (4)
C31	0.0170 (5)	0.0150 (5)	0.0164 (5)	−0.0023 (4)	0.0045 (4)	0.0006 (4)
N3	0.0173 (5)	0.0171 (5)	0.0175 (5)	−0.0011 (4)	0.0037 (4)	0.0016 (4)
C4	0.0226 (6)	0.0191 (6)	0.0212 (6)	−0.0050 (5)	0.0028 (5)	0.0023 (5)
C5	0.0210 (6)	0.0209 (6)	0.0287 (7)	0.0002 (5)	0.0049 (5)	0.0058 (5)
O5	0.0266 (5)	0.0285 (5)	0.0405 (6)	−0.0077 (4)	0.0084 (5)	0.0106 (5)
O1	0.0312 (5)	0.0226 (5)	0.0267 (5)	−0.0022 (4)	0.0148 (4)	0.0027 (4)
N2	0.0294 (6)	0.0205 (5)	0.0209 (5)	−0.0027 (5)	0.0105 (5)	0.0004 (4)
C32	0.0162 (5)	0.0166 (5)	0.0169 (5)	−0.0007 (4)	0.0029 (4)	−0.0007 (4)
N37	0.0173 (5)	0.0234 (5)	0.0171 (5)	−0.0047 (4)	0.0003 (4)	0.0017 (4)
P38	0.01456 (14)	0.01639 (14)	0.01647 (14)	−0.00207 (11)	0.00152 (11)	0.00093 (11)
C81	0.0151 (5)	0.0163 (5)	0.0213 (6)	−0.0010 (4)	0.0002 (4)	0.0015 (5)
C82	0.0191 (6)	0.0284 (7)	0.0265 (7)	−0.0044 (5)	0.0006 (5)	−0.0063 (6)
C83	0.0233 (6)	0.0269 (7)	0.0391 (8)	−0.0062 (6)	−0.0033 (6)	−0.0099 (6)
C84	0.0191 (6)	0.0218 (7)	0.0453 (9)	−0.0060 (5)	−0.0017 (6)	0.0023 (6)
C85	0.0242 (7)	0.0246 (7)	0.0365 (8)	−0.0034 (5)	0.0093 (6)	0.0052 (6)
C86	0.0256 (6)	0.0196 (6)	0.0272 (7)	−0.0030 (5)	0.0082 (5)	−0.0005 (5)
C61	0.0169 (5)	0.0172 (6)	0.0204 (6)	−0.0010 (4)	0.0040 (5)	0.0010 (4)
C62	0.0180 (6)	0.0210 (6)	0.0281 (7)	−0.0034 (5)	0.0036 (5)	−0.0023 (5)
C63	0.0236 (6)	0.0191 (6)	0.0376 (8)	−0.0019 (5)	0.0079 (6)	−0.0024 (6)
C64	0.0262 (7)	0.0224 (6)	0.0321 (7)	0.0054 (5)	0.0086 (6)	0.0054 (6)
C65	0.0247 (7)	0.0284 (7)	0.0254 (7)	0.0044 (6)	0.0001 (5)	0.0036 (5)
C66	0.0232 (6)	0.0219 (6)	0.0224 (6)	−0.0007 (5)	−0.0008 (5)	0.0003 (5)
C71	0.0159 (5)	0.0214 (6)	0.0164 (5)	−0.0024 (4)	0.0008 (4)	0.0010 (5)
C72	0.0211 (6)	0.0226 (6)	0.0243 (6)	0.0001 (5)	0.0051 (5)	0.0038 (5)
C73	0.0229 (6)	0.0262 (7)	0.0298 (7)	0.0025 (5)	0.0076 (6)	0.0006 (6)
C74	0.0209 (6)	0.0342 (7)	0.0194 (6)	−0.0022 (5)	0.0046 (5)	−0.0010 (5)
C75	0.0269 (7)	0.0349 (8)	0.0202 (6)	0.0010 (6)	0.0049 (5)	0.0086 (6)
C76	0.0239 (6)	0.0266 (7)	0.0231 (7)	0.0049 (5)	0.0046 (5)	0.0069 (5)
C33	0.0180 (6)	0.0205 (6)	0.0185 (6)	−0.0013 (5)	0.0024 (5)	0.0029 (5)
C34	0.0177 (6)	0.0195 (6)	0.0221 (6)	−0.0035 (5)	0.0043 (5)	0.0013 (5)

### Geometric parameters (Å, º)

**Table:**

Br35—C35	1.8945 (13)	C85—C86	1.387 (2)
C35—C36	1.3838 (18)	C85—H851	0.954
C35—C34	1.3892 (19)	C86—H861	0.934
C36—C31	1.3902 (17)	C61—C62	1.3959 (19)
C36—H361	0.945	C61—C66	1.3983 (19)
C31—N3	1.4371 (16)	C62—C63	1.389 (2)
C31—C32	1.4154 (17)	C62—H621	0.938
N3—C4	1.3381 (17)	C63—C64	1.386 (2)
N3—N2	1.3206 (16)	C63—H631	0.938
C4—C5	1.4101 (19)	C64—C65	1.389 (2)
C4—H41	0.908	C64—H641	0.935
C5—O5	1.2154 (17)	C65—C66	1.389 (2)
C5—O1	1.4101 (19)	C65—H651	0.928
O1—N2	1.3822 (15)	C66—H661	0.932
C32—N37	1.3598 (16)	C71—C72	1.3966 (19)
C32—C33	1.4148 (18)	C71—C76	1.3978 (18)
N37—P38	1.5698 (12)	C72—C73	1.393 (2)
P38—C81	1.8016 (13)	C72—H721	0.936
P38—C61	1.8062 (13)	C73—C74	1.386 (2)
P38—C71	1.8053 (13)	C73—H731	0.943
C81—C82	1.3885 (19)	C74—C75	1.385 (2)
C81—C86	1.4006 (19)	C74—H741	0.939
C82—C83	1.396 (2)	C75—C76	1.392 (2)
C82—H821	0.951	C75—H751	0.944
C83—C84	1.378 (2)	C76—H761	0.929
C83—H831	0.941	C33—C34	1.3850 (18)
C84—C85	1.385 (2)	C33—H331	0.935
C84—H841	0.927	C34—H341	0.920
			
Br35—C35—C36	119.37 (10)	C81—C86—C85	120.13 (14)
Br35—C35—C34	119.76 (10)	C81—C86—H861	119.7
C36—C35—C34	120.86 (12)	C85—C86—H861	120.2
C35—C36—C31	118.28 (12)	P38—C61—C62	118.47 (10)
C35—C36—H361	122.1	P38—C61—C66	121.98 (10)
C31—C36—H361	119.6	C62—C61—C66	119.55 (12)
C36—C31—N3	117.99 (11)	C61—C62—C63	120.13 (13)
C36—C31—C32	123.69 (11)	C61—C62—H621	119.9
N3—C31—C32	118.24 (11)	C63—C62—H621	120.0
C31—N3—C4	127.63 (11)	C62—C63—C64	120.12 (14)
C31—N3—N2	116.92 (11)	C62—C63—H631	120.3
C4—N3—N2	115.38 (11)	C64—C63—H631	119.6
N3—C4—C5	106.23 (12)	C63—C64—C65	120.08 (14)
N3—C4—H41	123.4	C63—C64—H641	119.3
C5—C4—H41	130.4	C65—C64—H641	120.6
C4—C5—O5	135.64 (15)	C64—C65—C66	120.20 (14)
C4—C5—O1	103.76 (11)	C64—C65—H651	120.3
O5—C5—O1	120.59 (13)	C66—C65—H651	119.5
C5—O1—N2	111.20 (10)	C61—C66—C65	119.92 (13)
O1—N2—N3	103.40 (11)	C61—C66—H661	119.2
C31—C32—N37	118.55 (11)	C65—C66—H661	120.8
C31—C32—C33	115.07 (11)	P38—C71—C72	118.53 (10)
N37—C32—C33	126.35 (12)	P38—C71—C76	121.79 (10)
C32—N37—P38	133.88 (10)	C72—C71—C76	119.49 (12)
N37—P38—C81	105.37 (6)	C71—C72—C73	119.60 (13)
N37—P38—C61	113.44 (6)	C71—C72—H721	120.7
C81—P38—C61	106.88 (6)	C73—C72—H721	119.7
N37—P38—C71	115.95 (6)	C72—C73—C74	120.53 (14)
C81—P38—C71	108.81 (6)	C72—C73—H731	118.4
C61—P38—C71	106.02 (6)	C74—C73—H731	121.0
P38—C81—C82	123.42 (10)	C73—C74—C75	120.17 (13)
P38—C81—C86	117.07 (10)	C73—C74—H741	122.0
C82—C81—C86	119.40 (12)	C75—C74—H741	117.8
C81—C82—C83	119.89 (14)	C74—C75—C76	119.79 (13)
C81—C82—H821	120.0	C74—C75—H751	119.4
C83—C82—H821	120.1	C76—C75—H751	120.8
C82—C83—C84	120.36 (14)	C71—C76—C75	120.40 (13)
C82—C83—H831	119.2	C71—C76—H761	121.1
C84—C83—H831	120.5	C75—C76—H761	118.5
C83—C84—C85	120.07 (14)	C32—C33—C34	122.22 (12)
C83—C84—H841	119.7	C32—C33—H331	119.2
C85—C84—H841	120.2	C34—C33—H331	118.6
C84—C85—C86	120.10 (14)	C35—C34—C33	119.87 (12)
C84—C85—H851	120.6	C35—C34—H341	119.4
C86—C85—H851	119.3	C33—C34—H341	120.8

### Hydrogen-bond geometry (Å, º)

**Table:**

*D*—H···*A*	*D*—H	H···*A*	*D*···*A*	*D*—H···*A*
C4—H41···O5^i^	0.91	2.48	3.344 (2)	159
C72—H721···O5^i^	0.94	2.37	3.297 (2)	173

Symmetry code: (i) −*x*+2, −*y*+2, −*z*+1.
